# Real-time detection of viable microorganisms by intracellular phototautomerism

**DOI:** 10.1186/1472-6750-10-45

**Published:** 2010-06-18

**Authors:** Remco Kort, Andreas Nocker, Alie de Kat Angelino-Bart, Sjaak van Veen, Herman Verheij, Frank Schuren, Roy Montijn

**Affiliations:** 1 Business Unit Food and Biotechnology Innovations, Microbial Genomics Group, TNO Quality of Life, Utrechtseweg 48, P.O. Box 360, 3700AJ Zeist, The Netherlands; 2 Business Unit Quality and Safety, Analytical Research Group, TNO Quality of Life, Utrechtseweg 48, P.O. Box 360, 3700AJ Zeist, The Netherlands; 3 Pyxis Discovery, Delftechpark 26, 2628 XH Delft, The Netherlands; 4 Top Institute Pharma, Galileiweg 8, 2333 BD Leiden, The Netherlands

## Abstract

**Background:**

To date, the detection of live microorganisms present in the environment or involved in infections is carried out by enumeration of colony forming units on agar plates, which is time consuming, laborious and limited to readily cultivable microorganisms. Although cultivation-independent methods are available, they involve multiple incubation steps and do mostly not discriminate between dead or live microorganisms. We present a novel generic method that is able to specifically monitor living microorganisms in a real-time manner.

**Results:**

The developed method includes exposure of cells to a weak acid probe at low pH. The neutral probe rapidly permeates the membrane and enters the cytosol. In dead cells no signal is obtained, as the cytosolic pH reflects that of the acidic extracellular environment. In live cells with a neutral internal pH, the probe dissociates into a fluorescent phototautomeric anion. After reaching peak fluorescence, the population of live cells decays. This decay can be followed real-time as cell death coincides with intracellular acidification and return of the probe to its uncharged non-fluorescent state. The rise and decay of the fluorescence signal depends on the probe structure and appears discriminative for bacteria, fungi, and spores. We identified 13 unique probes, which can be applied in the real-time viability method described here. Under the experimental conditions used in a microplate reader, the reported method shows a detection limit of 10^6 ^bacteria ml^-1^, while the frequently used LIVE/DEAD BacLight™ Syto9 and propidium iodide stains show detection down to 10^6 ^and 10^7 ^bacteria ml^-1^, respectively.

**Conclusions:**

We present a novel fluorescence-based method for viability assessment, which is applicable to all bacteria and eukaryotic cell types tested so far. The RTV method will have a significant impact in many areas of applied microbiology including research on biocidal activity, improvement of preservation strategies and membrane permeation and stability. The assay allows for high-throughput applications and has great potential for rapid monitoring of microbial content in air, liquids or on surfaces.

## Background

Ever since the pioneering work by Louis Pasteur and Robert Koch at the end of the nineteenth century, the detection of viable microorganisms has been carried out by cultivation and enumeration of colony forming units (CFU's). Almost all judgments on hygiene, food safety, drinking water quality, infections of pathogens, and efficacy of antimicrobials are based on growth on solid agar medium followed by CFU counts. However, the assessment of cell viability on agar plates is laborious, requires at least an overnight incubation, and is limited to microorganisms that are readily culturable under laboratory conditions [1]. These difficulties to directly measure the number of viable cells renders increasing importance to methods that measure indirect parameters (viability indicators) that live cells should possess. Such assays have been developed for the assessment of a variety of viability indicators, including membrane integrity [2], membrane potential [3,4], redox activity [5,6], ATP content [7,8], enzymatic activity [9,10], release of intracellular components [11], and presence of specific gene transcripts [12]. In this study, we present a novel and generic principle for the real-time viable (RTV) cell detection [13] based on intracellular phototautomerism that exclusively occurs in live cells exposed to a specific probe.

Phototautomerism [14] involves photo-excitation to the lowest excited singlet state, which results in the "simultaneous" loss of a proton from one moiety and gain of a proton by the other. This phenomenon, also known as excited state intramolecular proton transfer (ESIPT), is the very essence of the RTV assay reported here, as it results in a significant increase in fluorescence emission in the pH neutral cytoplasmic environment of live cells. Phototautomerism causes an anomalously large Stokes shift of fluorescence, in other words the excitation-emission shift is much higher than would be anticipated on the basis of the electronic structure of the neutral molecule, allowing fluorescence measurements with relatively high signal to background ratios.

The smallest and by far best-known phototautomeric compound described in this study is salicylic acid containing an aromatic hydroxyl as proton-donating and a carboxyl group as proton-accepting moiety. ESIPT in salicylic acid was first demonstrated by Weller [15], who noted that the fluorescence of salicylic acid occurred at much longer wavelength (~ 410 nm) than that of *o*-anisic acid (~ 340 nm), the latter containing an aromatic methoxyl group rather than a hydroxyl group, disabling the required intramolecular proton transfer.

Salicylic acid has been applied in a number of (bio)assays, including the detection of hydroxyl radicals by aromatic hydroxylation [16], the presence of Fe(III)-ions by complex formation [17], and the estimation of the intracellular pH by the use of radioactively labelled salicylate [18]. However, salicylic acid or other phototautomeric compounds have not been applied to date for fluorescence-based assessments of cell viability. The discovery of the novel application for salicylic acid reported here prompted us to screen *in vitro *for other phototautomeric compounds that efficiently permeate the cell membrane. This screening led to the identification of a number of probes, mostly aromatic carboxylic acids, which all show unique kinetic properties that greatly improve the versatility of the assay. Besides its ability to measure viability, the RTV assay shows different kinetics for fungi and bacteria and can be applied to spores, a major advantage over currently used methods. As the RTV assay rapidly discriminates between live and dead cells or organic material, the method holds great potential for rapid and high-throughput detection of microorganisms in air, in suspensions or on surfaces.

## Results

### Principles of the RTV assay

**Figure 1:**
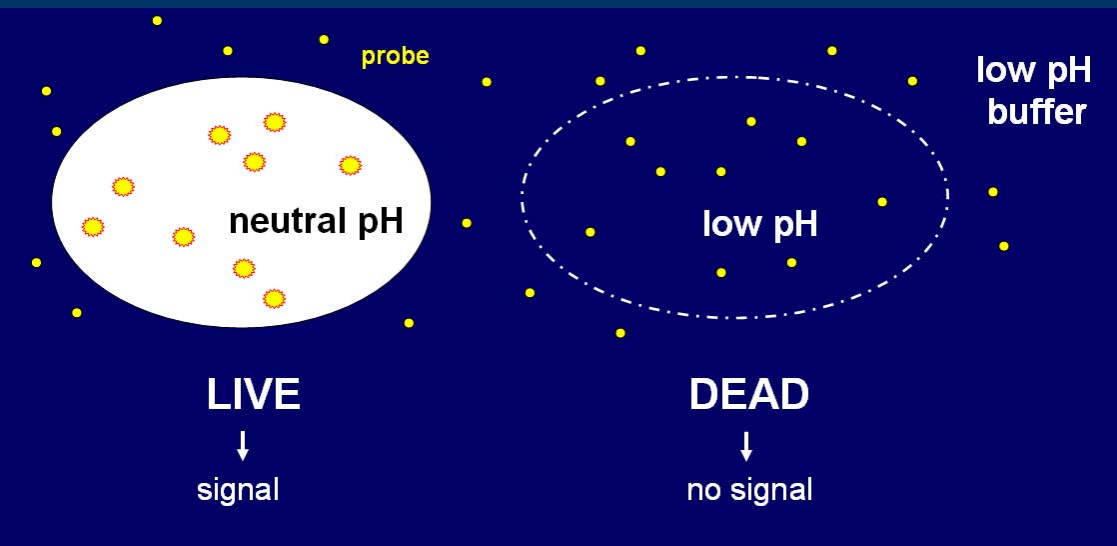
**Conceptual drawing of the RTV assay principle**. Live and dead cells are exposed to a probe in a low pH environment. The probe enters both cell types, but only emits a fluorescence signal in live cells which have maintained their neutral, intracellular pH. The pH in dead cells reflects the extracellular acidic conditions leading to signal suppression.

A schematic drawing of the assay principle is depicted in Figure 1. The uncharged probe is administered to live and dead cells in a low pH buffer and rapidly permeates the cell membranes independent of the cell's viability status [19]. The probe dissociates only in the neutral cytoplasmic environment of live cells to form an anion, which is highly fluorescent as a result of phototautomerism [14]. The probe does not dissociate in dead cells, where the cytoplasmic pH has equilibrated with the acidic environment.

**Figure 2:**
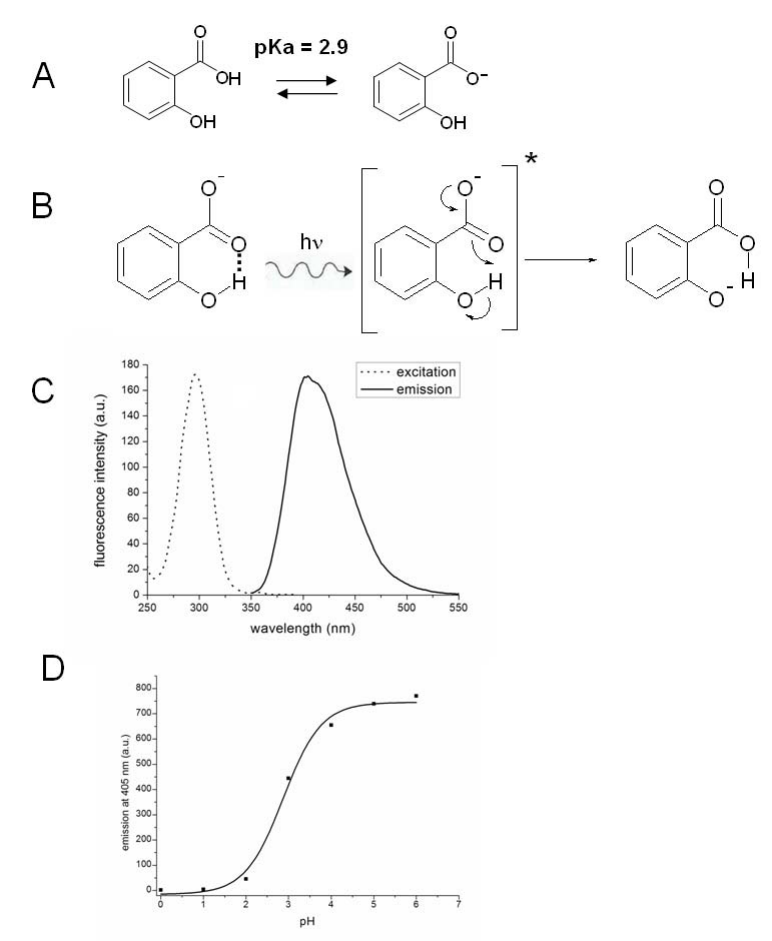
**Acid-base equilibrium and fluorescence properties of salicylic acid**. **(A) **Chemical structures of salicylic acid in its fully protonated form (at pH < pKa) and in its singly charged anion form (at pH > pKa). **(B) **Upon excitation, intramolecular electron rearrangements enable the proton which is associated with the phenolic group to be transferred to the carboxylic group, changing the fluorescence to that of the excited singly charged anion, ionized at the phenolic group; this phenomenon known as phototautomerism or excited state intramolecular proton transfer (ESIPT). **(C) **Fluorescence excitation and emission spectra of salicylic acid at pH 7.5. **(D) **Titration curve showing the pH-dependence of salicylic acid fluorescence emission.

The generic principles for the RTV assay are illustrated with salicylic acid as the model phototautomeric probe in Figure 2. Salicylic acid predominantly exists in the fully protonated form at pH values below the pKa value of its aromatic carboxylic group (pKa = 2.9). At pH values exceeding the pKa, this species converts into the singly charged anion (Figure 2A). Upon photoexcitation, the proton transfers intramolecularly from the phenolic group to the carboxylic group, changing the fluorescence emission to that of the excited phenolate anion (Figure 2B). This change results in an anomalously large Stokes shift of 115 nm (i.e. an excitation maximum at 295 nm and emission maximum at 410 nm; Figure 2C). As the fluorescence emission depends on the presence of the singly charged anion species, the signal intensity strongly depends on the pH (Figure 2D).

**Figure 3:**
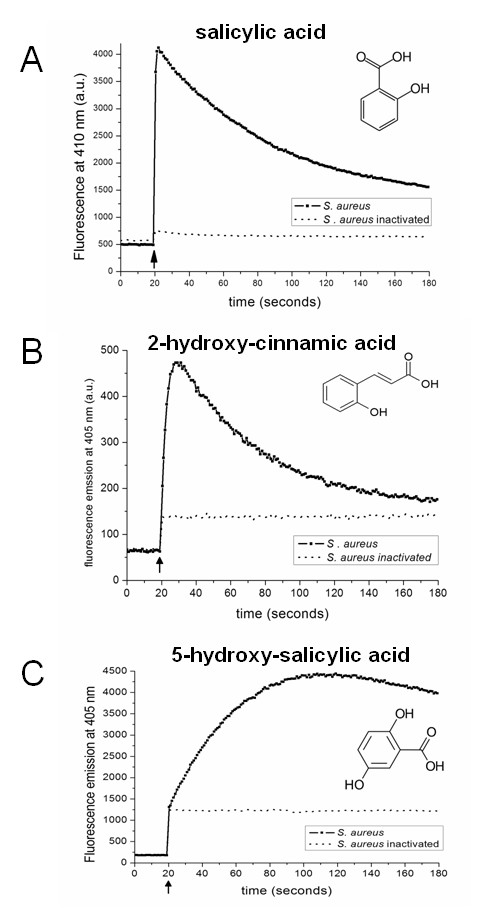
**Effect of phototautomeric probe structures on the RTV assay kinetics**. The RTV assay was performed with live and heat-killed *Staphylococcus aureus *cells using **(A) **salicylic acid, **(B) **2-hydroxy-cinnamic acid, and **(C) **5-hydroxy-salicylic acid as probes. Optimal excitation and emission properties were selected for the probes as outlined in table 1. Probes were dissolved in 100 mM phosphate buffer pH 2 at a concentration of 2 mM.

Accordingly, the probe can emit two types of fluorescence. At "low pH" the protonated acid is responsible for the fluorescence emission, whereas at "high pH" the phenolate-anion form is responsible for the fluorescence emission. Titration of this transition shows a pK of around 3 (Figure 2D). "High pH" conditions are typically found in the cytosol of living microbes where the intracellular pH is in the range between 5.6 and 9 [20,21]. Thus, if a membrane-permeable phototautomeric probe is added to living cells at low pH, the probe molecules will become fluorescent when entering the cells (Figure 3). The rapid membrane transfer of the probe goes along with intracellular acidification resulting from protons co-transferred with the weak acid probe, and (probe-enhanced) leakage of protons over the membrane. This continuous influx of protons along the pH gradient leads to a non-fluorescent "low pH" condition in the cell. In this way two parameters indicate the presence of living or viable cells: (1) the peak intensity of the fluorescence signal, and (2) the decay rate of the fluorescence of the cells in the acidic medium (Figure 3). The latter parameter reflects cytoplasmic acidification.

### Selection of phototautomeric probes

Identification of phototautomeric compounds with properties similar to salicylic acid was carried out by *in silico *screening and subsequent application of 3 different selection criteria (see Methods and Additional file 1 Tables S1 and S2). This led to the identification of 13 additional phototautomeric probes (compounds V2-V14, Table 1), none of which have, as far as we are aware, ever been applied as fluorescent probes for viability assessments or detection of live microorganisms. All of the selected phototautomeric compounds show an excitation maximum in the UV-region (ranging from 295-360 nm) and a relatively large Stokes shift in the range from 80 nm for 1-hydroxy-2-naphthoic acid (V6) to 160 nm for 5-amino salicylic acid (V3). In a next step we experimentally determined the fluorescence properties at acidic and neutral pH. For this purpose, we used the parameter pKf (Table 1), which represents the pH of the solution where both fluorescent species exist at the same concentration. Most of the selected phototautomeric compounds show an acidic pKf between pH 2 and pH 3, close to the pKa for aromatic carboxylic acids (Table 1). Notably, a large ratio between the fluorescence emission at high and low pH results in high signal to noise ratios for the assay. Salicylic acid (V1), 5-amino-salicylic acid (V3) and 7-amino-2,3-dihydro-benzo[1,4]dioxine-6-carboxylic acid (V13) show relatively high ratios ranging from 122 to 359. As a final probe selection criterion, we checked for efficient membrane permeation at low pH and identified phototautomeric compounds that permeate bacteria and yeast (V1, V6, V9), exclusively bacteria (V2, V4, V5, V11-V14), exclusively yeast (V10) or neither bacteria nor yeast (V3). Thus, the use of a combination of these compounds in one assay, whereby detection takes place at two or more wavelengths, allows discrimination of the above microorganisms.

**Table 1:** Selected probes for the RTV assay

code	name	exc. max. (nm)	em. max. (nm)	Stokes shift (nm)	pKf	pH ratio	uptake	
							
							*S. aur*	*S. cer*
V1	salicylic acid	295	405	110	2.9	122	+	+
V2	4-amino salicylic acid (PAS)	295 (265)	400	105	2.2 (3.5)	22 (74)	+	-
V3	5-amino salicylic acid (mesalamine)	335	495	160	5.8	331	-	-
V4	5-hydroxy salicylic acid (gentisic acid)	322	448	126	2.9	77	+	-
V5	4-hydroxy salicylic acid (β-resorcylic acid)	290	395	105	3.0	52	+	-
V6	1-hydroxy-2-naphtoic acid	340	420	80	3.0	3	+	+
V7	3-amino-2-naphtoic acid	360 (285)	470	110	4.5	88 (42)	+	+
V8	2-hydroxycinnamic acid (*o*-coumaric acid)	320 (360)	500	180	5.5 (>9)	30 (400)	+	-
V9	2-Hydroxy-dibenzofuran-3-carboxylic acid	340 (300)	450	110	2.3	4.3 (3.7)	+	+
V10	6-Amino-1,3-dimethyl-2-oxo-2,3-dihydro-1H-benzoimidazole-5-carboxylic acid	241	396	155	nd	1.4*	-	+
V11	5-(2-Ethyl-butyrylamino)-2-hydroxy-benzoic acid	311	426	115	nd	22.8*	+	-
V12	2-Hydroxy-5-[(tetrahydro-furan-2-carbonyl)-amino]-benzoic acid	307	417	110	nd	17*	+	-
V13	7-Amino-2,3-dihydro-benzodioxine-6-carboxylic acid	323	401	78	nd	359*	+	-
V14	2-Hydroxy-5-tetrazol-1-yl-benzoic acid	296	395	99	nd	5*	+	-

Overview of fluorescent compounds used for real-time monitoring of cell viability. Abbreviations: exc. max., excitation maximum; em. max., emission maximum; *S.aur*, *Staphyloccus aureus*; *S. cer, Saccharomyces cerevisiae*. The ratio of emission intensity was monitored at pH 7.5 and 1 as well as at pH 7 and 2 (indicated by the asterisk). Uptake of compounds by viable *S. aureus *and *S. cerevisiae *cells was monitored in a Tecan microplate fluorometer at optimal excitation and emission wavelengths.

### Assay kinetics for phototautomeric probes

The kinetics of the fluorescence signal depend on the nature of the probe being used. This is exemplified for *Staphylococcus aureus *(live and heat-killed cells) subjected to the RTV test using three different probes at a concentration of 25 μM at pH 2 (Figure 3). After addition of salicylic acid at t = 20 s, the signal maximum is reached very rapidly on a sub-second time scale (time constant k = 1.4 s^-1^) and subsequently decays relatively slowly (time constant k = 0.015 s^-1^) due to intracellular acidification (Figure 3A). The control sample containing heat-killed cells shows a small rise in fluorescence after probe addition resulting from fluorescent mono-anions present at pH 2 (Figure 3). The background obtained from samples with dead cells remains constant over time like the control sample without cells (not shown). Similar kinetics can be observed upon addition of the 2-hydroxy-cinnamic acid probe (Figure 3B). However, the fluorescence signal maximum is only about a tenth of that of salicylic acid, is reached more slowly than with salicylic acid, and occurs in a more confined collection of microbial species, as membrane transfer of this probe has not been observed in yeast cells (Table 1). Both the rise and decay rates of the fluorescent signal are lower than those observed for salicylic acid when the analogue 5-hydroxy salicylic acid is used as a probe (Figure 3C). As for salicylic acid, dead cells do not emit a rise and decay of the fluorescence signal with either of these probes.

### Assay kinetics for bacteria, fungi and spores

**Figure 4:**
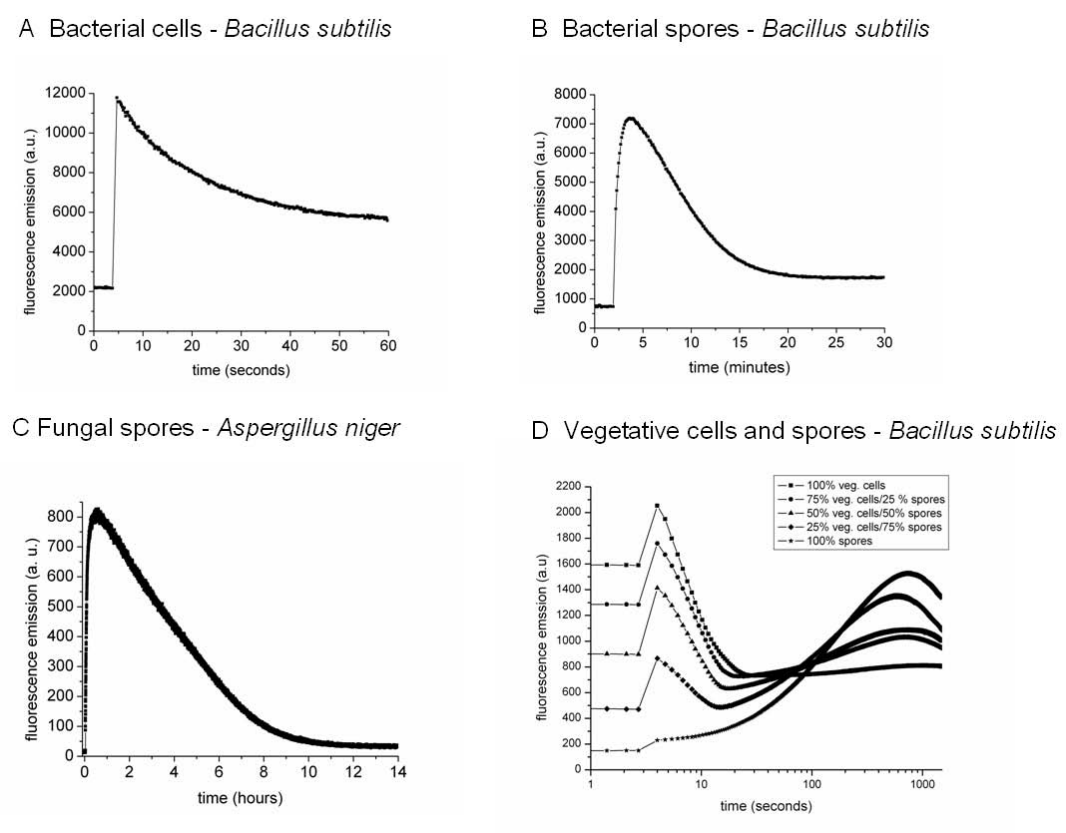
**RTV assay kinetics obtained with different microorganisms**. Salicylic acid (2 mM) was dissolved in 100 mM phosphate buffer and added to either live cells. Excitation was performed at 295 nm, emission at 405 nm. Fluorescence signals are shown for representatives of (**A**) bacterial cells at pH 2, **(B) **bacterial spores at pH 1 and **(C) **fungal spores at pH 1, and (**D**) a composite sample of *B. subtilis *spores and vegetative cells at pH 1.

In principle, the RTV assay can be carried out with any phototautomeric compound that fulfils the three criteria described above. To further substantiate the general applicability of the RTV assay we determined the kinetics of the fluorescence signal upon exposure of the salicylic acid probe to a wide range of microbial species. Besides the bacterium *Staphylococcus aureus *and the yeast *Saccharomyces cerevisiae *used for screening of the fluorescent probes, we have tested a variety of vegetative cells of Gram-positive bacteria, Gram-negative bacteria, yeasts, filamentous fungi, bacterial spores and fungal spores (data not shown). The RTV assay principles were successfully applied to each of these microorganisms, even to robust cells types like bacterial- and fungal spores. Notably, the kinetics of the assay change dramatically, depending on the type of microorganism and its physiological state. In general, the rate of the rise and decay of the fluorescence signal varies in the order bacteria > fungi > bacterial spores > fungal spores. An overview of fluorescence signals from three representative live microbial species is presented in Figure 4. The typical kinetics for 1 mM salicylic acid probe concentration, transferred into bacteria at pH 2, are represented by a rapid rise of the fluorescence signal on a sub-second time scale, followed by a decay varying from a few seconds to 1 minute; the fluorescence decay for the Gram-positive bacterium *Bacillus subtilis *(Figure 4A) was fit with a mono-exponential function with a time constant τ of 18 s. Under these conditions, probe fluorescence decay was very slow for bacterial and fungal spores. Therefore, we carried out the assay at pH 1 to accelerate the process. For the tested *B. subtilis *spore suspension, the fluorescence signal maximum was reached after approximately 2 min with a rise time τ of 24 s and the signal decay took place over a period of 20 min. (Figure 4B). The rise of the fluorescence signal took longer than 30 minutes for spores of the fungus *Aspergillus niger *(rise time τ of 240 s) and the decay more than 10 hours - the most robust cell type identified for the RTV assay described here (Figure 4C). The RTV assay on a composite test sample of vegetative cells and spores of *B. subtilis *shows that the assay kinetics are highly discriminative for the different cell types, taking place in approximately the first 10^2 ^seconds for vegetative cells and 10^3 ^seconds for bacterial spores (Figure 4D). Apparently, the time it takes to reach the maximum of fluorescence emission differs significantly among spore batches of of *B. subtilis *(compare 4B and 4D). This variability is attributed to differences in properties of the spore batches, possibly resulting from differences in the duration of storage in water at 4°C. The percentages of vegetative cells and spores were calculated, based on the peak maxima of fluorescence signals at the earliest time point of 1.3 s after probe injection for vegetative cells and ~ 600 s for this batch of spores. After background corrections, these calculations show good predictions for the numbers of viable cells and spores present, with errors ranging from 2 to 11% (data not shown).

### Detection limit

**Figure 5:**
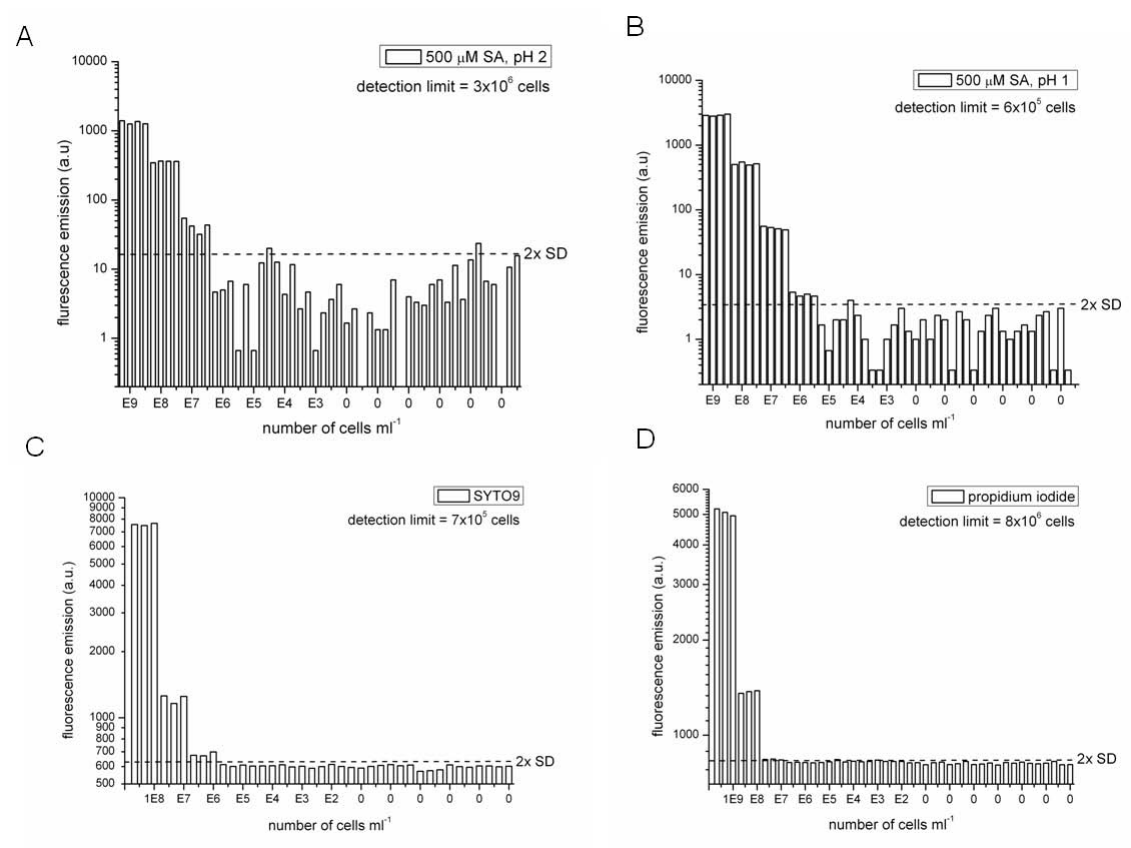
**The detection limit of the RTV assay and comparison to DEAD/LIVE BacLight™**. The detection limit was assessed for the salicylic acid dye of the RTV assay and the Syto9 and propidium iodide dyes of the DEAD/LIVE BacLight™ method [2] in the Infinite F500 Tecan microplate reader. Bars indicate the fluorescence intensities in arbitrary units at a range of cell concentrations. All assays were carried out with suspensions of *Staphylococcus aureus *bacteria diluted in physiological salt solution (PSS) ranging from 10^9 ^to 10^3 ^bacteria ml^-1 ^and were performed 6 times on PSS in the absence of cells to determine the average and 2× the standard deviation of the blank signal, as indicated by the dashed lines. **(A) **Measurements on live cells in quadruplicate, 500 μM salicylic acid, pH 2, excitation wavelength, 280 nm; emission wavelength, 400 nm **(B) **Measurements on live cells in quadruplicate, 500 μM salicylic acid, pH 1, excitation wavelength, 280 nm; emission wavelength, 400 nm **(C) **Measurements on live cells in triplicate (signals at 10^9 ^bacteria ml^-1 ^were omitted because of saturation), 10 μM Syto9 and 60 μM propidium iodide, pH 7 excitation wavelength, 485 nm; emission wavelength, 535 nm **(D) **Measurements on dead cells (heat-treated for 5 min at 95°C) in triplicate, 10 μM Syto9 and 60 μM propidium iodide, pH 7, excitation wavelength, 485 nm; emission wavelength, 612 nm.

The RTV assay was carried out with 2 mM, 500 μM and 50 μM of salicylic acid at pH 2 using a dilution series of a stationary phase culture of *Staphylococcus aureus*. The detection limit for RTV was defined here as the number of bacteria ml^-1 ^(extrapolated from a calibration curve) at twice the standard deviation of the blank signal (Figure 5, 2× SD, dashed lines), as determined by 24 assay measurements in the absence of bacteria (Figure 5A, B). The optimal concentration of salicylic acid was 500 μM, showing sufficient uptake of the dye and a minimum background signal. Interestingly, at pH 1 the detection limit significantly improves as result of reduced background fluorescence, which is below 20 arbitrary units at pH 2 and below 3 arbitrary units at pH 1, leading to detection limits of 3 × 10^6 ^bacteria ml^-1 ^and 6 × 10^5 ^bacteria ml^-1 ^(Figure 5A, B). This can be explained by the notion that at pH 1 the concentration of fluorescent anions of salicylic acid is significantly reduced (see also Figure 2D). The observed increase of the signal at high cell concentrations can be explained by the more efficient uptake of dye at pH 1. Clearly, the RTV assay allows detection of viable cells for at least 3 orders of magnitude, from 10^9 ^to 10^6 ^cells (Figure 5B). The background of fluorescence emission is relatively high (> 600) for Syto9 and propidium iodide dyes (Figure 5C, D). This background emission results from the fluorescence of these dyes in the absence of bacteria; the concentrations applied (10 μM Syto9 and 60 μM PI) were used as prescribed by the manufacturer; further optimization of the DEAD/LIVE BacLight™ assay could be achieved by lowering the concentrations of these dyes. This may also improve the linear range of this assay, as under the instrument settings applied, no read out was obtained for 10^9 ^bacteria ml^-1 ^for Syto9, resulting from detector saturation at this concentration of bacteria.

## Discussion

### Unique features

The RTV assay described here is based on fluorescence signals generated in living cells by intracellular phototautomerism of membrane-permeable probes. The increase in the fluorescence signal correlates with the number of living cells present. This fast and culture-independent assay can be performed in a high-throughput format. At least one of the phototautomeric probes reported here (salicylic acid) efficiently permeates bacterial and fungal spores. This unique trait of the assay has a large potential for applications, considering the current lack of cultivation-independent viability assays for these, often extremely resistant, cell types and the strong interest of industry for monitoring the viable spore load in different environments.

Salicylic acid turned out to be a generally applicable probe and was successfully applied to a number of different bacteria, fungi and spores. Whereas the signal maximum is reached almost instantaneously in bacteria, the kinetics of signal rise and decay are typically slower for fungi and much slower for bacterial and fungal spores. The uptake and decay rate kinetics thus appear unique for different microorganisms and offer the possibility to discriminate between bacteria, bacterial and fungal spores. The observed differences between the different cell types are not surprising considering that the probe uptake kinetics and fluorescence intensity are likely to depend on the intracellular pH, cell volume, water content, membrane composition and expression of specific uptake- and/or extrusion systems.

The information provided by RTV is twofold: The maximal signal intensity after probe addition and uptake by the cells correlates with the number of cells as well as with their viability status. In addition, RTV measures the ability of these live cells to maintain their cytosolic pH homeostasis when exposed to acidic conditions. The decay of the signal, which results from intracellular acidification, can be measured real-time and is a novel viability indicator.

### Mechanism

The phototautomeric probes used in this study permeate the cell membrane at relatively high rates in the range of a sub-second time scale for bacteria to the minute scale for spores. The efficient uptake can be explained by the relatively lipophilic nature of the probes and their net charge, as the entry of any molecule into a cell is governed by its lipid solubility according to the Overton Rule [22]. Diffusion of weak acids across membranes is further influenced by multiple proton transfer reactions in the aqueous layers adjacent to the membrane [23]. Such chemical reactions in unstirred layers adjacent to the membrane and their effects on the diffusion of solutes across that membrane were also demonstrated for salicylic acid [24]. Adsorption of salicylate anions to the lipid bilayer would increase a negative electrostatic surface potential, which would modify the interfacial ion concentrations and thus give rise to differences in transfer rates [25].

A model describing the possible mechanism of pH-dependent salicylic acid diffusion across membranes was presented previously [26]. It is in line with the diffusion of other weak acids like benzoic acid, acetic acid, lactic acid and sorbic acid. The efficient membrane permeation characteristics and their role as protonophores are the reason for the widespread use of these compounds as preservatives. Influx of these acids into cells and the accompanying proton transport across the cell membrane result in cytosolic acidification and uncoupling of the cellular proton motive force. The membrane permeation of these acids and their preservative effect is especially strong at an extracellular pH below the pKa of the acid. In contrast to the typical preservatives like benzoic acid or sorbic acid, the probes used in this study display pH-dependent phototautomerism allowing the monitoring of transfer over the membrane.

## Conclusions

**Figure 6:**
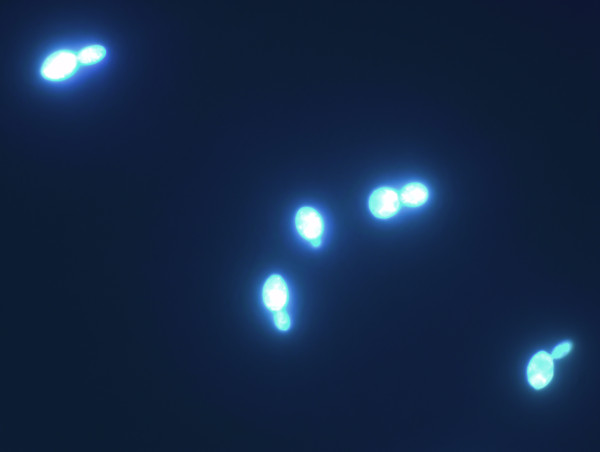
Fluorescence micrograph of *Saccharomyces cerevisiae *cells exposed to the RTV probe 1-hydroxy-2-naphtoic acid.

We present here a novel method for viability assessment, which is applicable to all bacteria (bacilli, enterobacteria, streptococci, etc.) and eukaryotic cell types (yeasts, filamentous fungi) tested so far. The RTV method is likely to have a significant impact in many areas of biology including research on (i) biocidal activity, (ii) improvement of preservation strategies, (iii) membrane permeation and stability. Based on our experience so far, the assay can successfully monitor stress and biocidal conditions affecting the cell membrane. However, we anticipate that for killing regimes that do not affect the membrane (UV, genotoxic compounds, or most antibiotics), the assay will not be predictive on short time scales after exposure. The assay reported here shows a detection limit of 10^6 ^bacteria ml^-1^, while the frequently used LIVE/DEAD BacLight™ Syto9 and propidium iodide stains show detection down to 10^6 ^and 10^7 ^bacteria ml^-1^, respectively. Immediate benefit of the assay was demonstrated in our lab for high-cell density applications, for example for assessing the viability of probiotic bacteria and starter cultures. In particular, the detection of viable bacterial and fungal spores is a unique and valuable feature of the assay. The assay can be performed very fast and in a high-throughput automated manner. The current drawback lies mainly in the limited sensitivity, and the requirement for environmental suspensions of relatively low optical density, a well-known limitation of fluorescence viability assays available. This problem might be overcome in the future by translation of the assay principles from a microplate fluorescence reader to flow cytometer-based or fluorescence microscopy-based platforms (Figure 6).

## Methods

### *In silico *screening of phototautomeric probes

In order to assess the exact requirements for the primary selection, three mechanisms [27] that have been identified for phototautomerism were first scrutinized: (i) (excited state) intramolecular proton transfer, (ii) simultaneous transfer of two protons, (iii) acid/base catalyzed proton transfer. Compounds were selected for which the presence of any one of these mechanisms for phototautomerism by performing extended (sub)structure searches in an in-house built "Global Supplier Database", consisting of 11 million commercially available compounds from approximately 200 suppliers (see http://www.pyxis-discovery.com). The 50 selected compounds are shown in Additional file 1 Table S1. In order to maximize the likelihood that only compounds would be tested that would display reasonable cell membrane permeation, cut-off values were used for both the calculated cLogP, a measure for the octanol/water partition coefficient, and cLogSW, the calculated water solubility at pH 7 (Additional file 1 Tables S1 and S2). Both these properties were calculated using CSpredict software from Chemsilico http://www.chemsilico.com. After the first round of screening, 9 compounds (V1 to V9; Table 1) were identified that displayed a relatively large Stokes shift, high pH dependence (ratio between fluorescence at pH 7/7.5 and pH 2/1), and were capable of permeating cells. The aim for the second round of screening was to improve fluorescence properties and cell permeation. Thus, 56 compounds were selected (Additional file 1 Table S2) using the same searching methodology and the same database that was used for the primary selection. Screening of these 56 compounds yielded an additional 5 compounds displaying favorable properties (V10 to V14; Table 1).

### *In vitro *screening of phototautomeric compounds

The selected compounds were screened *in vitro *by the following steps: (i) all compounds were dissolved in DMSO and diluted to 100 mM of phosphate buffers of pH 2 and pH 7 to a final concentration of 50 μM; (ii) excitation and emission maxima at pH 7 and pH 2 were determined by wavelength scans in the Tecan M200 fluorescence microplate reader, followed by selection of compounds with emission signal ratios at pH 7 and pH 2 of larger than 1; (iii) selected compounds were screened for permeation of *Staphylococcus aureas *ATCC 6538 and *Saccharomyces cerevisiae *cells ATCC 9763 as described for the RTV assay below.

### The RTV assay

Cells were grown overnight and were resuspended in 0.9% NaCl to reach an OD_600 _of 1.0. Aliquots of 100 μl of cell suspension were transferred into the wells of a 96-well microtiter plate (Greiner, flat bottom, UV-star plates) and inserted into the Tecan Infinite M200 plate reader. An identical volume (100 μl) of acidic probe solution was injected into the sample right before measurement. Probes were typically dissolved in 100 mM phosphate buffer pH 2 with a probe concentration of 2 mM. The plate reader settings for probe injection and measurement were as follows: injection mode; injection speed, 300 μl sec^-1^; manual gain, 100; number of flashes, minimal 10; bottom measurement; integration time, 20 μs; time interval, 1 s; total reading time, 180 s; probe injection at t = 20 seconds. Heat-inactivated cells (5 min at 95°C for vegetative cells) and compound and cell-free 0.9% NaCl solution served as controls. Measurements including salicylic acid were performed with excitation at 295 nm and emission reading at 405 nm. For other probes emission and excitation wavelength were selected based on the spectral properties as shown in Table 1.

The composite sample of bacterial spores and cells was prepared by addition of a suspension of *B. subtilis *spores of OD_600 _~ 1.5 to a suspension of vegetative cells of the same species of OD_600 _~ 1.5 in mixtures containing 0, 25, 50, 75 and 100% of spores (based on the OD_600 _values). The RTV assay was carried out as described above with 2 mM salicylic acid at pH 1 in the Infinite F500 Tecan microplate reader. All experiments were carried out in duplicate. For the calculations of percentages of spores and cells from the fluorescence signals, the peak maxima were determined, expressed in arbitrary fluorescence units and set to 100% for 100% spores and 100% vegetative cell-suspensions, respectively.

### The detection limit

The detection limit was assessed for the salicylic acid dye of the RTV assay and the Syto9 and propidium iodide (PI) dyes of BacLight™ method [2] with dilutions of cell suspensions (10^9 ^to 10^3 ^bacteria ml^-1^) of overnight cultures of *S. aureus *ATCC 6538 by fluorescence bottom readings in the Infinite F500 Tecan microplate reader. All measurements were carried out in triplicate or quadruplicate. The detection limit was determined by the number of bacteria ml^-1 ^in the calibration curve at twice the standard deviation of blank signals, as determined by 16-24 assay measurements in the absence of bacteria. The BacLight™ method was used according to the supplier's protocol. Briefly, volumes of 100 μl of live and heat-treated (5 min at 95°C) *S. aureus *cell suspensions were mixed with 100 μl of diluted BacLight™ dyes (10 μM Syto9 and 60 μM PI) and incubated for 15 min in the dark prior to the measurements. The RTV assay was carried out as described above for 2 mM, 500 μM and 50 μM of salicylic acid at pH 2 and pH 1. The plate reader settings were as follows for all dyes: manual gain, 50; number of flashes, minimal 10; integration time 20 μs; for salicylic acid: excitation wavelength, 280 nm; emission wavelength, 400 nm; excitation bandwidth, 20 nm; emission bandwidth, 25 nm; for Syto9 (live cells): excitation wavelength, 485 nm; emission wavelength, 535 nm; excitation bandwidth, 20 nm; emission bandwidth, 25 nm; and for PI (heat-treated cells): excitation wavelength, 485 nm; emission wavelength, 612 nm; excitation bandwidth, 20 nm; emission bandwidth, 10 nm.

## Authors' contributions

RK discovered the novel fluorescence-based method. RK and AN wrote the paper. RK, AN, SvV, HV and FHS participated in the experimental design and analyzed the data. AdKAB optimized the fluorescence measurements. HV carried out the *in silico *compound screening. RK, FHS and RCM participated in the coordination of the study. All authors read, corrected and approved the final manuscript.

## Supplementary Material

Additional file 1 **Table S1 Structures, names, and chemical properties of phototautomeric compounds analyzed in screening round 1 (50 compounds)**. Table S2 Screening round 2 (56 compounds). Click here for file

## References

[B1] Barer MR Harwood CR Bacterial viability and culturability Adv Microb Physiol 1999 41 93 137 full_text 10500845 10.1016/s0065-2911(08)60166-6

[B2] Boulos L Prevost M Barbeau B Coallier J Desjardins R LIVE/DEAD BacLight: application of a new rapid staining method for direct enumeration of viable and total bacteria in drinking water J Microbiol Methods 1999 37 77 86 10.1016/S0167-7012(99)00048-2 10395466

[B3] Mason DJ Allman R Lloyd D Lloyd D Uses of membrane potential dyes with bacteria Flow cytometry in microbiology 1993 London, United Kingdom: Springer-Verlag 67 81 pp. 67-81

[B4] Breeuwer P Abee T Assessment of the intracellular pH of immobilized and continuously perfused yeast cells employing fluorescence ratio imaging analysis J Microbiol Methods 2000 39 253 64 10.1016/S0167-7012(99)00124-4 10670771

[B5] Rodriguez GG Phipps D Ishiguro K Ridgway HF Use of a fluorescent redox probe for direct visualization of actively respiring bacteria Appl Environ Microbiol 1992 58 1801 8 1622256 10.1128/aem.58.6.1801-1808.1992 PMC195687

[B6] Tsukatani T Suenaga H Higuchi T Akao T Ishiyama M Ezoe K Matsumoto K Colorimetric cell proliferation assay for microorganisms in microtiter plate using water-soluble tetrazolium salts J Microbiol Methods 2008 75 109 16 10.1016/j.mimet.2008.05.016 18586343

[B7] Lundin A Use of firefly luciferase in ATP-related assays of biomass, enzymes, and metabolites Methods Enzymol 2000 305 346 70 full_text 10812612 10.1016/s0076-6879(00)05499-9

[B8] Petty RD Sutherland LA Hunter EM Cree IA Comparison of MTT and ATP-based assays for the measurement of viable cell number J Biolumin Chemilumin 1995 10 29 34 10.1002/bio.1170100105 7762413

[B9] Stubberfield LCF Shaw PJA A comparison of tetrazolium reduction and FDA hydrolysis with other methods of microbial activity J Microbiol Methods 1990 12 151 162 10.1016/0167-7012(90)90026-3

[B10] Hoefel D Grooby WL Monis PT Andrews S Saint CP A comparative study of carboxyfluorescein diacetate and carboxyfluorescein diacetate succinimidyl ester as indicators of bacterial activity J Microbiol Methods 2003 52 379 88 10.1016/S0167-7012(02)00207-5 12531507

[B11] Kort R O'Brien AC van Stokkum IH Oomes SJ Crielaard W Hellingwerf KJ Brul S Assessment of heat resistance of bacterial spores from food product isolates by fluorescence monitoring of dipicolinic acid release Appl Environ Microbiol 2005 71 3556 64 10.1128/AEM.71.7.3556-3564.2005 16000762 PMC1169001

[B12] Kort R Keijser BJ Caspers MP Schuren FH Montijn R Transcriptional activity around bacterial cell death reveals molecular biomarkers for cell viability BMC Genomics 2008 9 590 10.1186/1471-2164-9-590 19061518 PMC2648990

[B13] Kort R Schuren F Montijn R Real-time method for the detection of viable microorganisms Patent WO2009082218 2009

[B14] Schulman SG Wehry EL Acid-Base Chemistry of Excited Singlet States Modern Fluorescence Spectroscopy 1976 London: Heyden 263 266 pp. 263-266

[B15] Weller AH Fast reactions of excited molecules Prog React Kinet 1961 1 187 214

[B16] Kaur H Halliwell B Detection of hydroxyl radicals by aromatic hydroxylation Methods Enzymol 1994 233 67 82 full_text 8015497 10.1016/s0076-6879(94)33009-3

[B17] Cha KW Park KW Determination of iron(III) with salicylic acid by the fluorescence quenching method Talanta 1998 46 1567 71 10.1016/S0039-9140(98)00032-0 18967288

[B18] Garcia-Sancho J Sanchez A Use of salicylic acid to measure the apparent intracellular pH in the Ehrlich ascites-tumor cell and Escherichia coli Biochim Biophys Acta 1978 509 148 58 10.1016/0005-2736(78)90015-9 25670

[B19] Thomae AV Wunderli-Allenspach H Kramer SD Permeation of aromatic carboxylic acids across lipid bilayers: the pH-partition hypothesis revisited Biophys J 2005 89 1802 11 10.1529/biophysj.105.060871 15951388 PMC1366683

[B20] Bakker EP The role of alkali-cation transport in energy coupling of neutrophilic and acidophilic bacteria: an assessment of methods and concepts FEMS Microbiol Rev 1990 75 319 334

[B21] Booth IR Regulation of cytoplasmic pH in bacteria Microbiol Rev 1985 49 359 78 3912654 10.1128/mr.49.4.359-378.1985 PMC373043

[B22] Al-Awqati Q One hundred years of membrane permeability: does Overton still rule? Nat Cell Biol 1999 1 E201 2 10.1038/70230 10587658

[B23] Antonenko YN Denisov GA Pohl P Weak acid transport across bilayer lipid membrane in the presence of buffers. Theoretical and experimental pH profiles in the unstirred layers Biophys J 1993 64 1701 10 10.1016/S0006-3495(93)81542-X 8369403 PMC1262505

[B24] Gutknecht J Tosteson DC Diffusion of Weak Acids across Lipid Bilayer Membranes: Effects of Chemical Reactions in the Unstirred Layers Science 1973 182 1258 1261 10.1126/science.182.4118.1258 4752218

[B25] McLaughlin S Salicylates and Phospholipid Bilayer Membranes Nature 1973 243 234 236 10.1038/243234a0 4706296

[B26] Takagi M Taki Y Sakane T Nadai T Sezaki H Oku N Yamashita S A new interpretation of salicylic acid transport across the lipid bilayer: implications of pH-dependent but not carrier-mediated absorption from the gastrointestinal tract J Pharmacol Exp Ther 1998 285 1175 80 9618420

[B27] Chou PT The Host/Guest Type of Excited-State Proton Transfer; A General Review J Chin Chem Soc 2001 48 651 82

